# Oxoberberine: a promising natural antioxidant in physiological environments†

**DOI:** 10.1039/d2ra01372j

**Published:** 2022-03-29

**Authors:** Pham Cam Nam, Nguyen Quang Trung, Nguyen Thi Hoa, Huynh Ngoc Bich, Tran Duc Manh, Duong Tuan Quang, Adam Mechler, Quan V. Vo

**Affiliations:** Department of Chemical Engineering, The University of Danang-University of Science and Technology Danang 550000 Vietnam; The University of Danang-University of Science and Education Da Nang 550000 Vietnam; The University of Danang - University of Technology and Education Danang 550000 Vietnam vvquan@ute.udn.vn; Hue University Hue 530000 Vietnam; Department of Chemistry and Physics, La Trobe University Victoria 3086 Australia

## Abstract

Oxoberberine (OB, 2,10-dihydroxy-3,9-dimethoxy-8-oxo-protoberberine, artathomsonine), which was isolated from Artabotrys thomsonii, was shown to exhibit potent antioxidant activity *in vitro*, however that is the only reported evidence of the radical scavenging activity of this compound thus far. In the present study, thermodynamic and kinetic calculations were used to determine the free radical scavenging activity of OB against a range of biologically important species, under physiological conditions. In the first part the activity is calculated against the HOO˙ radical that is both biologically important and a reference radical for comparison. It was found that OB has high antiradical capacity against HOO˙ in both lipid medium and water at physiological pH with *k*_overall_ = 1.33 × 10^5^ and 1.73 × 10^6^ M^−1^ s^−1^, respectively. The formal hydrogen transfer mechanism defined the activity in nonpolar environments, whereas in the aqueous solution the single electron transfer competes with the hydrogen transfer pathway. The results showed that, in lipid medium, the HOO˙ trapping capability of OB is better than typical antioxidants such as Trolox, BHT, resveratrol and ascorbic acid. Similarly, the activity of OB in water at pH 7.4 is roughly 19 and 7 times faster than those of Trolox and BHT, respectively, but slightly lower than the activities of resveratrol or ascorbic acid. In the second part, it was found that OB also exhibits high activity against other typical free radicals such as CH_3_O˙, CH_3_OO˙, CCl_3_OO˙, NO_2_, SO_4_˙^−^, DPPH and ABTS˙^+^ with *k*_f_ ranging from 2.03 × 10^5^ to 5.74 × 10^7^ M^−1^ s^−1^. Hence, it is concluded that OB is a promising radical scavenger in the physiological environment.

## Abstract 2

Oxoberberine (OB), a radical scavenger discovered from *Artabotrys thomsonii*, is expected to be effective in physiological environments.

## Graphical abstract

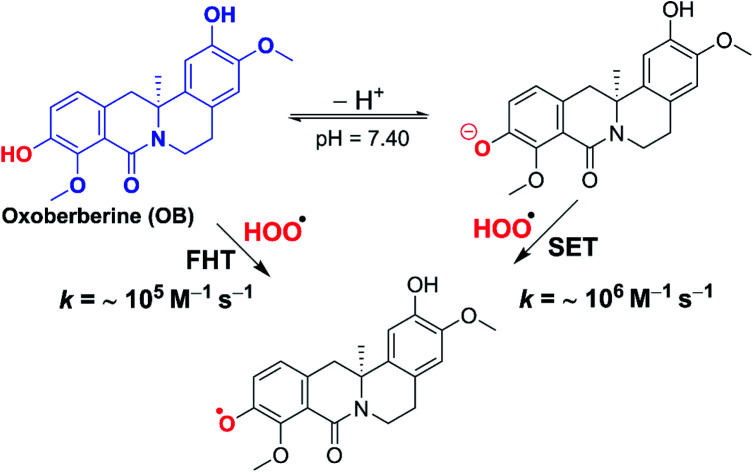

## 1. Introduction

**Fig. 1:**
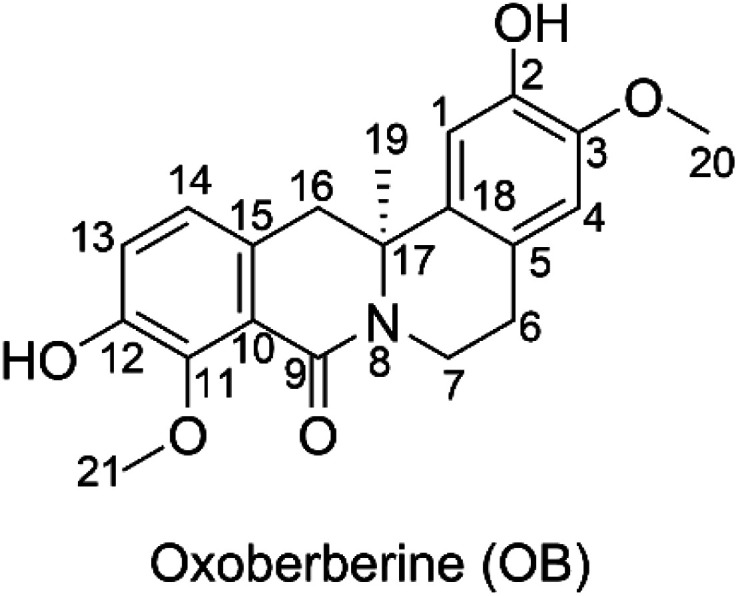
Chemical structure of oxoberberine (OB).

The custard-apple family Annonaceae (Juss.) includes the genus *Artabotrys*, which is one of the largest genera.^1^ The genus *Artabotrys* contains traditional medicinal plants used for the treatment of a variety of diseases, including malaria, scrofula, cholera, diabetes, stomach pain, asthma, and cough.^2–4^ The search for the key active ingredients yielded oxoberberine (OB, 2,10-dihydroxy-3,9-dimethoxy-8-oxo-protoberberine, artathomsonine, Fig. 1), which was isolated from *Artabotrys thomsonii*.^1^ This compound belongs to the berberine family, which has recently attracted attention due to its confirmed biological activities, including antidiabetic,^5^ anticancer,^6,7^ antimicrobial,^8,9^ and antioxidant properties.^10^ The latter is based on the berberine structure with two phenolic groups (Fig. 1) that would normally impose antioxidant properties on natural products. OB exhibited good antioxidant activity in the ferric reduction ability potential (FRAP) assay with 0.2 ± 0.05 μg gallic acid equivalents per mg compound,^1^ but this is the only evidence thus far for its antioxidant activity.

Oxidative stress (OS) is a chemical term that refers to an imbalance in the synthesis and consumption of oxidants in biological systems.^11^ Despite the presence of oxidants of various chemical natures in such systems, free radicals (FR) stand out in the OS environment. They are highly reactive and have the ability to initiate chain reactions, leading to spreading molecular damage.^12^ There are several different types of FR, the majority of which can be divided into two categories: reactive oxygen species (ROS, *i.e.* HO˙, CH_3_O˙, HOO˙, CH_3_OO˙, O_2_˙^−^, SO_4_˙^−^) and reactive nitrogen species (RNS, *i.e.* NO, NO_2_, 
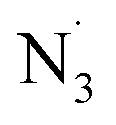
). The hydroxyl radical has been blamed for the majority of ionizing radiation-induced DNA oxidation and tissue damage,^13–15^ whereas peroxynitrite is a potent oxidant and a very toxic species that can damage lipids, proteins, and DNA when it reacts with O_2_˙^−^.^16,17^ The HOO˙ radical is considered a model free radical to evaluate the antiradical activity of organic compound due to its moderate reactivity,^12,18^ while studies on the radical scavenging activity against other typical ROS and RNS such as HO˙, CH_3_O˙, CCl_3_O˙, HOO˙, CH_3_OO˙, CCl_3_OO˙, NO, NO_2_, O_2_˙^−^, SO_4_˙^−^ and 
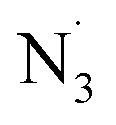
 are crucial to provide practical information about the antioxidant activity of natural products.^19,20^

The benefit of computational approaches to the analysis of structure–activity relationship has been demonstrated in a number of previous studies, making it a valuable addition to the medicinal chemistry toolbox.^12,21–27^ In this study, we used computational methods to analyze the radical scavenging activity of OB in physiological environments against HO˙, CH_3_O˙, CCl_3_O˙, HOO˙, CH_3_OO˙, CCl_3_OO˙, NO, NO_2_, O_2_˙^−^, SO_4_˙^−^, 
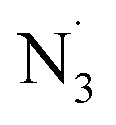
, DPPH and ABTS˙^+^.

## 2. Computational details

The Gaussian 09 suite of programs^28^ was used to perform all calculations in this study by using the density functional theory (DFT) approach. All computations were performed using the M06-2X functional and the 6-311++G(d,p) basis set.^29^ The M06-2X functional is one of the most dependable approaches for studying radical reaction thermodynamics and kinetics.^30,31^ With only modest inaccuracies when compared to experimental data (*k*_calc_/*k*_exp_ ratio = 1–2.9),^18,32–35^ and widely applied to evaluate the radical scavenging activity of both natural and artificial compounds.^36–39^ The SMD technique,^36^ which is often employed when modelling the radical scavenging activity of antioxidants,^30,35,37^ was utilized to predict the solvent effects of water and pentyl ethanoate.

The kinetic calculations were performed using the quantum mechanics-based test for overall free radical scavenging activity (QM-ORSA) protocol.^18^ Standard transition state theory (TST) and a 1 M standard state at 298.15 K were used to compute the rate constants (*k*).^35,38–44^ More details on the method can be found in Table S1, ESI.†

## 3. Results and discussion

### 3.1. Conformer evaluation

**Fig. 2:**
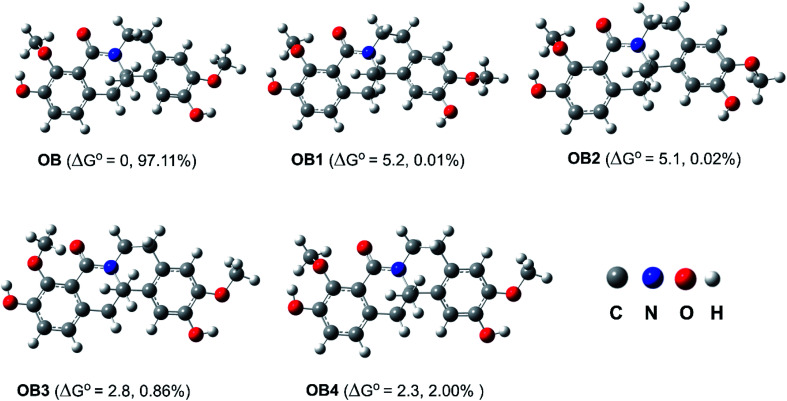
The main conformers of OB (Δ*G*^o^ (in kcal mol^−1^) compared with OB).

The OH and OMe groups in OB can rotate around the single bonds to generate a variety of conformers, according to research on the OB structure. Thus, the probable OB conformers were screened^45^ in the first stage, and the five lowest electronic energy conformers were then examined using the M06-2X/6-311++G(d,p) level of theory (Fig. 2). Among the conformers, OB has the lowest Gibbs free energy value while OB1–OB4 have higher free energy of formation than OB by 2.3–5.2 kcal mol^−1^. When the relative populations of the conformers were estimated using the Maxwell–Boltzmann distribution,^46,47^ it was found that OB is dominant (>97%), hence this conformer was studied in the followings.

### 3.2. The HOO radical scavenging activity of OB

#### 3.2.1. The thermodynamic study

Thermodynamic calculations based on the three common radical scavenging mechanisms were used to assess antioxidant activity. These are (i) formal hydrogen transfer (FHT), (ii) single electron transfer-proton transfer (SETPT), and (iii) sequential proton-loss electron transfer (SPLET).^48^ The HOO˙ radical quenching of OB in physiological environments (pentyl ethanoate for a lipid medium and water at pH 7.4) was examined first by calculating the thermodynamic parameters (bond dissociation enthalpy (BDE), ionization energy (IE), proton affinity (PA) and the Gibbs free energy change (Δ*G*^o^) of the first step of each mechanism) for all bonds. Table 1 summarizes the findings.

**Table 1:** The computed thermodynamic parameters (BDE, PA, IE in kcal mol^−1^) of OB and Δ*G*^o^ (kcal mol^−1^) of the first step of the HOO˙ + OB reaction in the studied solvents

Positions	Pentyl ethanoate						Water					
	BDE	Δ*G*^o^	PA	Δ*G*^o^	IE	Δ*G*^o^	BDE	Δ*G*^o^	PA	Δ*G*^o^	IE	Δ*G*^o^
C6–H	88.4	1.5			132.0	69.3	88.9	1.2			107.0	26.3
C7–H	92.8	6.4					94.2	4.5				
C16–H	88.9	2.4					89.8	1.1				
O2–H	86.5	0.2	87.4	106.5			88.0	−1.6	48.2	56.5		
O12–H	87.2	0.2	86.4	105.3			87.7	−2.6	44.9	52.8		

It was found that BDE values in the lipid medium range from 86.5 to 92.8 kcal mol^−1^, which are 0.5–1.5 kcal mol^−1^ lower than those in the aqueous solution. The IE values are larger than the BDEs in all of the studied solvents. This suggests that the single electron transfer (SET) pathway is not feasible in either of the environments. However, the deprotonation at the O2(12)–H bond could preferentially occur in water due to lower PA values compared with the BDEs and IEs (PA = 44.9–48.2 kcal mol^−1^).

The OB + HOO^−^ reaction was only spontaneous in the thermodynamic sense following the hydrogen transfer pathway, particularly at the O2(12)–H bonds (Δ*G*^o^ = −2.6 to 0.2 kcal mol^−1^). In polar media such as water at pH = 7.40, the deprotonation and stable states of OB should also be considered.

#### 3.2.2. The kinetic study

**Fig. 3:**
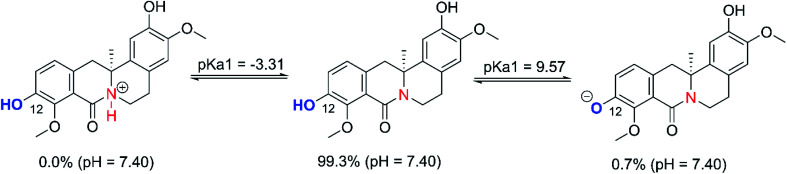
Possible protonation states of OB at pH = 7.40.

The protonation state of OB at physiological pH must be assessed in order to evaluate the feasibility and kinetics of electron transfer processes from the deprotonated species. The OB structure allows protonation at the N8 site (1), as well as deprotonation of the alcohol molecule at the O12–H position (2). Following the literature, the p*K*_a_ values of OB were computed based on the model reactions (1) and (2), the results are presented in Fig. 3.^49,50^

1 R_2_NH^+^ ⇆ R_2_N + H^+^

2 ROH ⇆ RO^−^ + H^+^

**Fig. 4:**
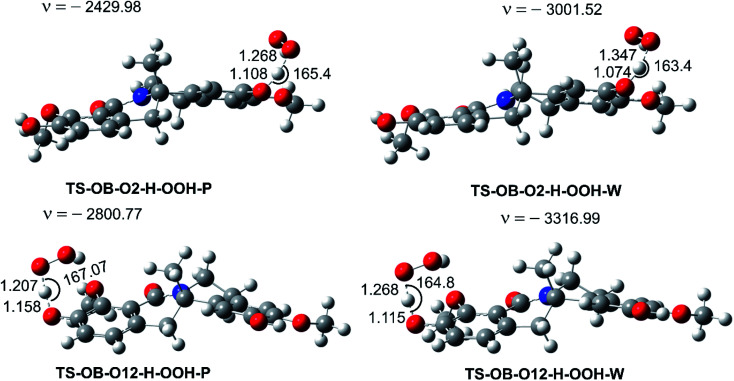
The selected TS structures of the HOO˙ + OB reaction following the FHT pathway (P: pentyl ethanoate, W: water).

The computed p*K*_a_ values for the amine were <1, while the O12–H group had a p*K*_a_ of 9.57. As a consequence, the cationic state is not relevant but there is still a non-negligible proportion of the mono-anionic state (O12–H, 0.7%) in a pH 7.4 aqueous solution (Fig. 3). Thus, both the neutral and anionic states were considered in the kinetic evaluation of HOO˙ antiradical activity of OB in water at pH of 7.4. eqn (3) and (4) can be used to assess the total rate constant (*k*_overall_) of OB's antiradical behavior against HOO˙ radical in the physiological environments. The results are presented in Table 2 and Fig. 4.

**Table 2:** Computed Δ*G*^‡^ in kcal mol^−1^, tunneling correction (*κ*), *Γ* in %, and *k*_app_, *k*_f_, and *k*_overall_ in M^−1^ s^−1^ of OB + HOO˙ reactions^a^

Mechanisms		Pentyl ethanoate				Water					
		Δ*G*^‡^	*κ*	*k*_app_	*Γ*	Δ*G*^‡^	*κ*	*k*_app_	*f*	*k*_f_	*Γ*
SET						6.2	15.7	1.80 × 10^8^	0.007	1.26 × 10^6^	73.0
FHT	O2–H	17.3	2459.8	3.30 × 10^3^	2.5	15.5	7917.2	2.00 × 10^5^	0.993	1.99 × 10^5^	11.5
	O12–H	16.2	15 234	1.30 × 10^5^	97.5	17.3	199 934	2.70 × 10^5^	0.993	2.68 × 10^5^	15.5
*k*_overall_				1.33 × 10^5^						1.73 × 10^6^	

a *k*_f_ = *f*. *k*_app_; *Γ* = *k*.100/*k*_overall_; the nuclear reorganization energy (*λ*, in kcal mol^−1^).

Lipid environment:

3 *k*_overall_ = *k*_app_(FHT(O2–H)-neutral) + *k*_app_(FHT(O12–H)-neutral)

Water at physiological pH:

4 *k*_overall_ = *k*_f_(SET-anion) + *k*_f_(FHT(O2–H)-neutral) + *k*_f_(FHT(O12–H)-neutral)

In lipid media, the H-abstraction of the O12–H bond dominates in the HOO˙ radical scavenging of OB with a rate constant of *k* = 1.30 × 10^5^ M^−1^ s^−1^, whereas in aqueous solution, SPLET was the main mechanism with *k* = 1.26 × 10^6^ M^−1^ s^−1^ (Δ*G*^‡^ = 6.2 kcal mol^−1^, Δ*G*^o^ = 4.0 kcal mol^−1^). The FHT reaction of the O2–H bond contributes about 11.5% of the overall rate constant in the aqueous solution, and only = 2.5% (*k* = 3.30 × 10^3^ M^−1^ s^−1^) in the lipid medium. Overall the HOO˙ antiradical activity of OB in the polar environment is approximately 13 times faster than in the lipid medium (*k*_overall_ = 1.73 × 10^6^ M^−1^ s^−1^*vs. k*_overall_ = 1.33 × 10^5^ M^−1^ s^−1^, respectively).

Therefore, in lipid medium, the HOO˙ trapping capability of OB is higher than that of typical antioxidants such as Trolox (*k*_overall_ = 3.40 × 10^3^ M^−1^ s^−1^),^33^ BHT (*k*_overall_ = 1.70 × 10^4^ M^−1^ s^−1^),^51^ resveratrol (*k*_overall_ = 1.31 × 10^4^ M^−1^ s^−1^)^37^ and ascorbic acid (*k*_overall_ = 5.71 × 10^3^ M^−1^ s^−1^).^18^ In water at physiological pH, OB is about 13 and 7 times more active than Trolox (*k* = 8.96 × 10^4^ M^−1^ s^−1^)^33^ and BHT (*k*_overall_ = 2.51 × 10^5^ M^−1^ s^−1^),^51^ respectively, but slightly less active than resveratrol (*k* = 5.62 × 10^7^ M^−1^ s^−1^),^37^ ascorbic acid (*k* = 9.97 × 10^7^ M^−1^ s^−1^),^18^ cannabidiolic acid (*k* = 2.40 × 10^6^ M^−1^ s^−1^),^52^ or carnosic acid (*k* = 4.73 × 10^6^ M^−1^ s^−1^).^53^ Hence, OB is a promising radical scavenger in physiological environments.

### 3.3. The antiradical activity of OB against ordinary free radicals in aqueous solution

Whereas HOO˙ scavenging activity is useful measure for comparison, there are variances in radical scavenging activities against different radical species. Therefor next the antiradical activity of OB was modeled against a range of common free radicals such as HO˙, CH_3_O˙, CCl_3_O˙, HOO˙, CH_3_OO˙, CCl_3_OO˙, NO, NO_2_, O_2_˙^−^, SO_4_˙^−^, 
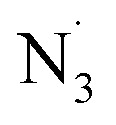
, DPPH and ABTS˙^+^ that are used in experimental antiradical assays. The antiradical activity of OB against these free radicals may follow either of the typical mechanisms such as FHT, SETPT, SPLET^48^ or/and radical adduct formation (RAF),^12^ however, the study showed that the SET mechanism plays a determining role in the hydroperoxyl radical scavenging activity of OB (*Γ* = 73.0%). Thus in order to rationalize computing time and for comparability with previous data,^19,20^ the interaction of the anion state with these radicals was examined following the principal aqueous phase mechanism (the SET reaction) at pH = 7.4, with the results presented in Table 3.

**Table 3:** Calculated kinetic data between OB-O12-ANION and the selected radicals

Radical	Δ*G*^‡^	*λ*	*k*_app_	*k*_f_ a
HO˙	13.5	3.8	7.90 × 10^2^	5.53
CH_3_O˙	0.7	4.9	8.20 × 10^9^	5.74 × 10^7^
CCl_3_O˙	14.6	21.6	1.20 × 10^2^	8.40 × 10^−1^
HOO˙	6.2	15.7	1.80 × 10^8^	1.26 × 10^6^
CH_3_OO˙	7.3	15.1	2.90 × 10^7^	2.03 × 10^5^
CCl_3_OO˙	0.0	17.2	6.90 × 10^9^	4.83 × 10^7^
NO	92.6	14.7	8.40 × 10^−56^	5.88 × 10^−58^
NO_2_	1.1	28.1	8.20 × 10^9^	5.74 × 10^7^
O_2_˙^−^	51.1	17.5	2.00 × 10^−25^	1.40 × 10^−27^
SO_4_˙^−^	6.6	18.0	8.90 × 10^7^	6.23 × 10^5^
N_3_˙	14.7	2.8	1.00 × 10^2^	7.00 × 10^−1^
DPPH	4.1	19.2	3.50 × 10^9^	2.45 × 10^7^
ABTS˙^+^	1.6	12.2	6.60 × 10^9^	4.62 × 10^7^

a *k*_f_ = *f*. *k*_app_; *f*(A^−^) = 0.007.

According to the calculations, OB should have high activity against CH_3_O˙, CH_3_OO˙, CCl_3_OO˙, NO_2_, SO_4_˙^−^ radicals with *k*_f_ ranging from 2.03 × 10^5^ to 5.74 × 10^7^ M^−1^ s^−1^, whereas HO˙, CCl_3_O˙, NO, O_2_˙^−^ and 
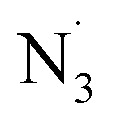
 radicals could not be removed under the examined conditions. The results also suggest that OB can exhibit the significant DPPH and ABTS˙^+^ radicals scavenging activity (*k*_f_ = 2.45 × 10^7^ and 4.62 × 10^7^ M^−1^ s^−1^, respectively) in water at pH = 7.40. Compared with fraxin^19^ and usnic acid,^20^OB exhibited the lower HO˙, CCl_3_O˙, 
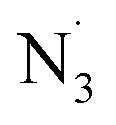
 antiradical activity than that of these compounds by the SET reaction, whereas the opposite trend was observed at peroxyl radicals *i.e.* HOO˙, and CH_3_OO˙.

## 4. Conclusion

Computer calculations were used to evaluate the radical scavenging capacity of oxoberberine against HO˙, CH_3_O˙, CCl_3_O˙, HOO˙, CH_3_OO˙, CCl_3_OO˙, NO, NO_2_, O_2_˙^−^, SO_4_˙^−^, 
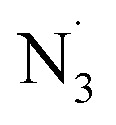
, DPPH and ABTS˙^+^. In the physiological environment OB exhibited high antiradical capacity. Concerning the reference system HOO˙, the overall rate constant of the radical scavenging of OB was 1.73 × 10^6^ and 1.33 × 10^5^ M^−1^ s^−1^ in the aqueous solution and lipid medium, respectively. The FHT mechanism defined the activity in nonpolar solvents, whereas that for water at pH = 7.40 had contributions from the SPLET as well as FHT pathways. It was also found that OB exhibits high antiradical activity against CH_3_O˙, CH_3_OO˙, CCl_3_OO˙, NO_2_, SO_4_˙^−^, DPPH and ABTS˙^+^ with *k*_f_ ranging from 2.03 × 10^5^ to 5.74 × 10^7^ M^−1^ s^−1^. The calculated results showed that the HOO˙ trapping capability of OB is also higher than those of typical antioxidants such as Trolox and BHT in both the lipid and polar environments.

## Conflicts of interest

There are no conflicts to declare.

## Supplementary Material

RA-012-D2RA01372J-s001
