# Supplementary material for: Oxoberberine: a promising natural antioxidant in physiological environments
Source: RSC Adv. 2022 Mar 29;12(16):9738–43. doi: 10.1039/d2ra01372j (PMC8961270; doi:10.1039/d2ra01372j)
Supplement: RA-012-D2RA01372J-s001 [file RA-012-D2RA01372J-s001.pdf]

Electronic Supplementary Material (ESI) for RSC Advances.
This journal is © The Royal Society of Chemistry 2022

Supporting Information (SI)

Oxoberberine: a promising natural antioxidant in physiological environments

Pham Cam Nam,[1] Nguyen Quang Trung,[2] Nguyen Thi Hoa,[3] Huynh Ngoc Bich,[3] Tran Duc Manh,[2] Duong Tuan Quang,[4] Adam Mechler[5] and Quan V. Vo[3*]

[1]Department of Chemical Engineering, The University of Danang-University of Science and Technology, Danang 550000, Vietnam

[2]The University of Danang-University of Science and Education, Da Nang 550000, Vietnam

[3]The University of Danang - University of Technology and Education, Danang 550000, Vietnam.

[4]Hue University, Hue 530000, Vietnam

[5]Department of Chemistry and Physics, La Trobe University, Victoria 3086, Australia.

Corresponding authors: vvquan@ute.udn.vn

Table of Contents

Table S1. The method to calculate rate constant following the conventional transition state theory S2

Table S2: The Cartesian coordinates and energies of TS of the reaction between compound OB with $HOO^{\bullet}$ following the FHT mechanism in studied solvents S4

References S8

Table S1. The method to calculate rate constant following the conventional transition state theory

The rate constant (k) was calculated by using the conventional transition state theory (TST) (at 298.15 K, 1M standard state) according to the equation (1):[1-5]

$$k = \sigma\kappa \frac{k_B T}{h} e^{-(\Delta G^{\neq})/RT} \quad (1)$$

Where: σ is the reaction symmetry number ,[6,7]

κ contains the tunneling corrections calculated using the Eckart barrier,[8]

k_B is the Boltzmann constant,

h is the Planck constant,

$\Delta G^{\neq}$ is the Gibbs free energy of activation.

The Marcus Theory was used to estimate the reaction barriers of SET reactions.[9-12] The free energy of reaction $\Delta G^{\neq}$ for the SET pathway was computed following the equations (2,3).

$$\Delta G^{\neq}_{SET} = \frac{\lambda}{4}\left(1 + \frac{\Delta G^{0}_{SET}}{\lambda}\right)^2 \quad (2)$$

$$\lambda \approx \Delta E_{SET} - \Delta G^{0}_{SET} \quad (3)$$

where ΔG_{SET} is the Gibbs energy of reaction, ΔE_{SET} is the non-adiabatic energy difference between reactants and vertical products for SET.[13,14]

For rate constants that were close to the diffusion limit a correction was applied to yield realistic results[15]. The apparent rate constants (k_{app}) were calculated following the Collins–Kimball theory in the solvents at 298.15K;[16] the steady-state Smoluchowski rate constant (k_D) for an irreversible bimolecular diffusion–controlled reaction was calculated following the literature as corroding to equations (4,5).[15,17]

$$k_{app} = \frac{k_{TST} k_D}{k_{TST} + k_D} \quad (4)$$

$$k_D = 4\pi R_{AB} D_{AB} N_A \quad (5)$$

where R_{AB} is the reaction distance, N_A is the Avogadro constant, and $D_{AB} = D_A + D_B$ (D_{AB} is the mutual diffusion coefficient of the reactants A and B),[16,18] where D_A or D_B is estimated using the Stokes–Einstein formulation (6).[19,20]

$$D_{A\ or\ B} = \frac{k_B T}{6\pi \eta a_{A\ or\ B}} \quad (6)$$

η is the viscosity of the solvents (i.e. $\eta(H_2O) = 8.91\times10^{-4}$ Pa s, η(pentyl ethanoate) = 8.62×10^{-4} Pa s) and *a* is the radius of the solute.

The kinetic study requires different considerations. In this study, the solvent effects of water and pentyl ethanoate were modelled by the solvation model density (SMD) method [21,22]. Water (dielectric constants, ε = 78.35) and pentyl ethanoate (ε = 4.73) are the *de facto* standard solvents in the literature to mimic the polar and nonpolar environments in the human body [15,23-25]. Thus, these solvents were used to model the physiological environments. The solvent cage effects were included following the corrections proposed by Okuno,[26] adjusted with the free volume theory according to the Benson correction[15,27-29] to reduce over-penalizing entropy losses in solution. For the species that have multiple conformers, all of these were investigated and the conformer with the lowest electronic energy was included in the analysis.[24,25] The hindered internal rotation treatment was also applied to the single bonds to ensure that the obtained conformer has the lowest electronic energy [25,30]. All transition states were characterized by the existence of only one single imaginary frequency. Intrinsic coordinate calculations (IRCs) were performed to ensure that each transition state is connected correctly with the pre-complex and post-complex.

Table S2: The Cartesian coordinates and energies of TS of the reaction between compound OB with HOO$^{\bullet}$ following the FHT mechanism in studied solvents

Name	OB-C2-H-OOH (pentyl ethanoate)
Cartesian Coordinates	Frequency and Energy
C 4.02297000 0.31378500 -0.62894000<br>C 3.47670800 -0.98530500 -0.39582700<br>C 2.12564400 -1.08457800 -0.02635400<br>C 1.30390900 0.02713500 0.07075600<br>C 1.85891000 1.29967800 -0.17091600<br>C 3.20786800 1.42839000 -0.51387900<br>C -0.17578300 -0.19038900 0.40406900<br>N -0.88942600 1.10203600 0.42598800<br>C -0.14491100 2.25431500 0.91795400<br>C 1.01374200 2.53455000 -0.02245400<br>C -0.79132800 -1.03548900 -0.72121300<br>C -2.27903500 -1.15285500 -0.55739800<br>C -2.98434000 -0.02824900 -0.10896700<br>C -2.23553700 1.23004200 0.21953200<br>C -2.95480000 -2.33546300 -0.83190400<br>C -4.32903300 -2.43023900 -0.64811600<br>C -5.03187600 -1.32592100 -0.19083100<br>C -4.37138700 -0.11510300 0.06290700<br>O -2.79979000 2.31230600 0.30292400<br>O 5.33882900 0.34399800 -0.92473400<br>C 5.91229100 1.59824200 -1.29369400<br>O 4.19803200 -2.06655400 -0.57964000<br>O -5.16407200 0.88825800 0.54824300<br>C -5.62060200 1.80977000 -0.45215400<br>O -6.36762300 -1.41788900 0.01952900<br>C -0.31688500 -0.87470400 1.77289600<br>H 1.74900200 -2.08593000 0.15089600<br>H 3.60674900 2.42183900 -0.67863400<br>H -0.82634400 3.09769700 0.97681000<br>H 0.23163600 2.03582900 1.92369100<br>H 1.62362300 3.35865000 0.35492900<br>H 0.61919300 2.83662100 -0.99923000<br>H -0.55385300 -0.55507200 -1.67725100<br>H -0.33661200 -2.02711700 -0.72646800<br>H -2.39846100 -3.19941200 -1.17845200<br>H -4.86254300 -3.35396100 -0.83950600<br>H 6.94758100 1.38541900 -1.54969800<br>H 5.39490000 2.01549600 -2.16048800<br>H 5.87635100 2.30470900 -0.46003300<br>H 5.09077900 -2.07190900 0.08136000<br>H -6.25269200 2.53152100 0.06309300<br>H -4.77333700 2.32010000 -0.90715700<br>H -6.20547900 1.28411000 -1.21404500<br>H -6.64852600 -0.60069200 0.45514400<br>H 0.11463200 -0.24549700 2.55429500<br>H -1.37055400 -1.04514000 2.00374900<br>H 0.20001400 -1.83593000 1.78517800<br>H 5.85057200 -0.11711200 1.06691100<br>O 5.89803000 -1.97135800 1.05448900<br>O 5.52537000 -0.81302800 1.66421300	Zero-point correction= 0.397197 (Hartree/Particle)<br>Thermal correction to Energy= 0.422695<br>Thermal correction to Enthalpy= 0.423639<br>Thermal correction to Gibbs Free Energy= 0.341955<br>Sum of electronic and zero-point Energies= -1356.025847<br>Sum of electronic and thermal Energies= -1356.000349<br>Sum of electronic and thermal Enthalpies= -1355.999405<br>Sum of electronic and thermal Free Energies= -1356.081089

**Name**	**OB-C2-H-OOH (water)**
Cartesian Coordinates	Frequency and Energy
C 4.04355100 0.27687100 -0.70037400	Zero-point correction= 0.396085 (Hartree/Particle)
C 3.47840500 -1.00874800 -0.45468000	Thermal correction to Energy= 0.421785
C 2.13261200 -1.10401400 -0.08323500	Thermal correction to Enthalpy= 0.422729
C 1.32800900 0.02009800 0.01970200	Thermal correction to Gibbs Free Energy= 0.340119
C 1.90182000 1.28794600 -0.21418200	Sum of electronic and zero-point Energies= -1356.031279
C 3.24794600 1.40549100 -0.56316600	Sum of electronic and thermal Energies= -1356.005579
C -0.14958800 -0.18204600 0.36520100	Sum of electronic and thermal Enthalpies= -1356.004635
N -0.85835900 1.11834700 0.37801500	Sum of electronic and thermal Free Energies= -1356.087244
C -0.09745000 2.26668600 0.86660300	
C 1.07174700 2.53100300 -0.06213600	
C -0.78004900 -1.03963000 -0.73970900	
C -2.26542500 -1.14457300 -0.55829600	
C -2.96006800 -0.01734200 -0.10158600	
C -2.20064700 1.23521500 0.21270400	
C -2.95142300 -2.32225800 -0.82999600	
C -4.32405700 -2.40762300 -0.63478200	
C -5.01578900 -1.30031100 -0.16762600	
C -4.34624000 -0.09779700 0.08537300	
O -2.77273100 2.32480400 0.31617200	
O 5.34561500 0.27554000 -1.03093800	
C 5.99645600 1.54088200 -1.16880500	
O 4.22169800 -2.10049500 -0.61070300	
O -5.11811400 0.92870200 0.57523600	
C -5.66587500 1.76778700 -0.45625600	
O -6.36102900 -1.39590700 0.05205800	
C -0.28150700 -0.84482400 1.74495600	
H 1.74562100 -2.10013900 0.10064600	
H 3.65977700 2.39417900 -0.72284300	
H -0.76011200 3.12405300 0.92080300	
H 0.26632200 2.04381600 1.87511700	
H 1.68922400 3.34173700 0.32966400	
H 0.69716700 2.84802800 -1.04143300	
H -0.55177600 -0.57925900 -1.70733800	
H -0.33475300 -2.03494000 -0.73045800	
H -2.40251700 -3.18808200 -1.18258500	
H -4.86516000 -3.32718900 -0.82486900	
H 7.03521200 1.31476700 -1.39443600	
H 5.55278900 2.11164600 -1.98665200	
H 5.92949900 2.10431100 -0.23548700	
H 5.02284700 -2.08447400 0.10454000	
H -6.22869700 2.54968300 0.05001600	
H -4.86290000 2.20787800 -1.04742000	
H -6.33274900 1.18713300 -1.09939300	
H -6.66075900 -0.58062500 0.48055500	
H 0.16880500 -0.21064100 2.51119100	
H -1.33381000 -1.00001000 1.99208100	
H 0.22356100 -1.81197400 1.75510900	
H 5.60617700 -0.02980600 1.43230500	
O 5.75912900 -1.88070900 1.21403700	
O 5.14670300 -0.81261000 1.78620400	
**Name**	**OB-C12-H-OOH (pentyl ethanoate)**
Cartesian Coordinates	Frequency and Energy

C 5.04590700 -0.18688700 -0.06364800<br>C 4.35655200 -1.37426700 0.23696600<br>C 2.98843000 -1.34827700 0.40290300<br>C 2.26481800 -0.15197700 0.27482700<br>C 2.94934800 1.02574500 -0.01418000<br>C 4.34283200 0.99747500 -0.18135100<br>C 0.74143900 -0.22327700 0.41968900<br>N 0.14661900 1.11747000 0.23690900<br>C 0.93106300 2.26029200 0.69285200<br>C 2.21449300 2.33855600 -0.11276800<br>C 0.20559000 -1.12122300 -0.70866200<br>C -1.29094000 -1.11116600 -0.75425000<br>C -1.94838600 0.08786900 -0.45501600<br>C -1.14199200 1.32107000 -0.15752800<br>C -2.02094400 -2.26431600 -1.07233700<br>C -3.39717500 -2.24453700 -1.07400600<br>C -4.09183500 -1.05630200 -0.76131200<br>C -3.34354500 0.11471000 -0.43614600<br>O -1.62413200 2.43808800 -0.28314400<br>O 6.39018000 -0.34202300 -0.20115900<br>C 7.15604600 0.81239500 -0.51504300<br>O 5.03790500 -2.54148600 0.36508800<br>O -4.04841600 1.20022000 -0.01531600<br>C -4.54508700 2.05336300 -1.05934700<br>O -5.39927700 -1.01969200 -0.79113000<br>C 0.35528800 -0.76327700 1.80598300<br>H 2.49190600 -2.28436800 0.63243100<br>H 4.86094900 1.92490700 -0.39641200<br>H 0.32271400 3.15250900 0.57662200<br>H 1.16368600 2.12726200 1.75547900<br>H 2.84416400 3.14784000 0.26494500<br>H 1.97158000 2.56906000 -1.15567500<br>H 0.60380900 -0.74972300 -1.66055300<br>H 0.56654100 -2.14201000 -0.57618300<br>H -1.48742200 -3.17968500 -1.30285800<br>H -3.98071000 -3.12737900 -1.30761900<br>H 8.18911200 0.47974800 -0.58892100<br>H 6.83934600 1.23953300 -1.47038200<br>H 7.06885900 1.56309600 0.27522700<br>H 5.97522100 -2.36025000 0.21626100<br>H -5.11645200 2.83617600 -0.56436900<br>H -3.70480400 2.48992000 -1.59851100<br>H -5.19406000 1.48477200 -1.72867800<br>H -5.83848300 -0.69361300 0.22930500<br>H 0.73221300 -0.09475000 2.58303900<br>H -0.73099900 -0.83120600 1.90232300<br>H 0.77832300 -1.75520700 1.97415400<br>H -4.59903100 0.50613600 1.64978000<br>O -6.11557200 -0.53073000 1.39287100<br>O -4.91746500 -0.34448500 2.01109600	Zero-point correction= 0.396708 (Hartree/Particle)<br>Thermal correction to Energy= 0.422380<br>Thermal correction to Enthalpy= 0.423325<br>Thermal correction to Gibbs Free Energy= 0.340884<br>Sum of electronic and zero-point Energies= -1356.027024<br>Sum of electronic and thermal Energies= -1356.001352<br>Sum of electronic and thermal Enthalpies= -1356.000408<br>Sum of electronic and thermal Free Energies= -1356.082848
**Name**	**OB-C12-H-OOH (water)**
Cartesian Coordinates	Frequency and Energy
C 5.03391100 -0.20406300 -0.07282000<br>C 4.32910400 -1.39165400 0.18045500	Zero-point correction= 0.395444 (Hartree/Particle)<br>Thermal correction to Energy= 0.421465

C	2.96080000	-1.36168200	0.34218100	Thermal correction to Enthalpy= 0.422409
C	2.24935300	-0.15461200	0.25466200	Thermal correction to Gibbs Free Energy= 0.338555
C	2.94801300	1.02555300	0.01227500	Sum of electronic and zero-point Energies= -1356.027598
C	4.34143400	0.99054100	-0.14953700	Sum of electronic and thermal Energies= -1356.001577
C	0.72656600	-0.21829100	0.39940000	Sum of electronic and thermal Enthalpies= -1356.000633
N	0.14028800	1.13344000	0.23914000	Sum of electronic and thermal Free Energies= -1356.084488
C	0.93455100	2.25582900	0.73915200	
C	2.22981700	2.34908400	-0.04260600	
C	0.17969000	-1.09651400	-0.73629000	
C	-1.31504500	-1.07191200	-0.77183900	
C	-1.96115100	0.13063900	-0.46097300	
C	-1.13979700	1.34491100	-0.14386100	
C	-2.05254400	-2.21868300	-1.09782600	
C	-3.42801200	-2.19085500	-1.08898500	
C	-4.10657000	-1.00040700	-0.76281400	
C	-3.35698600	0.16974500	-0.44304100	
O	-1.61900900	2.47864200	-0.24373300	
O	6.38232400	-0.35710700	-0.21258800	
C	7.14721800	0.81629200	-0.47529200	
O	4.99794400	-2.58511100	0.27013000	
O	-4.05364200	1.26349200	-0.03418600	
C	-4.62541900	2.04405400	-1.10045100	
O	-5.42614300	-0.96010800	-0.76114400	
C	0.34036000	-0.77622800	1.77741300	
H	2.45460000	-2.30093900	0.53616100	
H	4.86891700	1.92026900	-0.32683500	
H	0.34418000	3.16211200	0.65065500	
H	1.14861900	2.08064100	1.79861800	
H	2.86121500	3.13226700	0.38243800	
H	2.01259200	2.62829600	-1.07869400	
H	0.57110300	-0.72008500	-1.68880900	
H	0.52930800	-2.12206500	-0.61478500	
H	-1.52341500	-3.13345600	-1.33835400	
H	-4.01838700	-3.06896900	-1.32256000	
H	8.18006200	0.48762100	-0.56192100	
H	6.82701600	1.28216800	-1.41005200	
H	7.05340000	1.52864100	0.34762600	
H	5.94129700	-2.42160000	0.13193200	
H	-5.16208300	2.86026700	-0.62225100	
H	-3.82845500	2.43828100	-1.73224500	
H	-5.31391900	1.43504200	-1.68770800	
H	-5.80039500	-0.70913700	0.25889900	
H	0.73265200	-0.12946400	2.56478500	
H	-0.74641700	-0.82968800	1.87779900	
H	0.75002000	-1.77810700	1.91496900	
H	-4.41192000	0.35998500	1.84857700	
O	-5.96685100	-0.61467500	1.51299900	
O	-4.69759400	-0.56096300	1.99884100	

References

1. M. G. Evans and M. Polanyi, *Trans. Faraday Soc.*, 1935, **31**, 875-894.
2. H. Eyring, *J. Chem. Phys.*, 1935, **3**, 107-115.
3. D. G. Truhlar, W. L. Hase and J. T. Hynes, *J. Phys. Chem.*, 1983, **87**, 2664-2682.
4. T. Furuncuoglu, I. Ugur, I. Degirmenci and V. Aviyente, *Macromolecules*, 2010, **43**, 1823-1835.
5. E. Vélez, J. Quijano, R. Notario, E. Pabón, J. Murillo, J. Leal, E. Zapata and G. Alarcón, *J. Phys. Org. Chem.*, 2009, **22**, 971-977.
6. E. Pollak and P. Pechukas, *J. Am. Chem. Soc.*, 1978, **100**, 2984-2991.
7. A. Fernández-Ramos, B. A. Ellingson, R. Meana-Pañeda, J. M. Marques and D. G. Truhlar, *Theor. Chem. Acc.*, 2007, **118**, 813-826.
8. C. Eckart, *Phy. Rev.*, 1930, **35**, 1303.
9. R. A. Marcus, *Annu. Rev. Phys. Chem.*, 1964, **15**, 155-196.
10. R. A. Marcus, *Rev. Mod. Phys.*, 1993, **65**, 599.
11. Y. Lu, A. Wang, P. Shi and H. Zhang, *PloS one*, 2017, **12**, e0169773.
12. Y. Lu, A. Wang, P. Shi, H. Zhang and Z. Li, *PloS one*, 2015, **10**, e0133259.
13. S. F. Nelsen, S. C. Blackstock and Y. Kim, *J. Am. Chem. Soc.*, 1987, **109**, 677-682.
14. S. F. Nelsen, M. N. Weaver, Y. Luo, J. R. Pladziewicz, L. K. Ausman, T. L. Jentzsch and J. J. O'Konek, *J. Phys. Chem. A*, 2006, **110**, 11665-11676.
15. A. Galano and J. R. Alvarez-Idaboy, *J. Comput. Chem.*, 2013, **34**, 2430-2445.
16. F. C. Collins and G. E. Kimball, *J. Colloid Sci.* , 1949, **4**, 425-437.
17. M. Von Smoluchowski, *Z. Phys. Chem*, 1917, **92**, 129-168.
18. D. G. Truhlar, *J. Chem. Educ.*, 1985, **62**, 104.
19. A. Einstein, *Ann. Phys.*, 1905, **17**, 549-560.
20. G. G. Stokes, *Mathematical and Physical Papers*, University Press, Cambridge, 1905.
21. A. V. Marenich, C. J. Cramer and D. G. Truhlar, *The Journal of Physical Chemistry B*, 2009, **113**, 6378-6396.
22. C. P. Kelly, C. J. Cramer and D. G. Truhlar, *Journal of chemical theory and computation*, 2005, **1**, 1133-1152.
23. A. Galano and J. Raúl Alvarez-Idaboy, *Int. J. Quantum Chem.*, 2019, **119**, e25665.
24. Q. V. Vo, T. V. Gon, M. V. Bay and A. Mechler, *J. Phys. Chem. B*, 2019, **123**, 10672-10679.
25. Q. V. Vo and A. Mechler, *J. Chem. Inf. Model.*, 2020, **60**, 316-321.
26. Y. Okuno, *Chem.: Eur. J.*, 1997, **3**, 212-218.
27. S. Benson, *The foundations of chemical kinetics:* , Malabar, Florida, 1982.
28. C. Iuga, J. R. Alvarez-Idaboy and A. Vivier-Bunge, *J. Phys. Chem. B*, 2011, **115**, 12234-12246.

29. J. R. Alvarez-Idaboy, L. Reyes and N. Mora-Diez, *Org. Biomol. Chem.*, 2007, **5**, 3682-3689.
30. T. H. Le, T. T. Tran and L. K. Huynh, *Chemom. Intell. Lab. Syst.*, 2018, **172**, 10-16.
